# ‘Pitfalls for differentiating basal cell carcinoma from non‐basal cell carcinoma on optical coherence tomography: A clinical series’

**DOI:** 10.1111/1346-8138.17020

**Published:** 2023-11-06

**Authors:** Tom Wolswijk, Patty J. Nelemans, Fieke Adan, Myrurgia Abdul Hamid, Klara Mosterd

**Affiliations:** ^1^ Department of Dermatology Maastricht University Medical Center+ Maastricht The Netherlands; ^2^ GROW Research Institute for Oncology and Reproduction Maastricht University Maastricht The Netherlands; ^3^ Department of Epidemiology Maastricht University Maastricht The Netherlands; ^4^ Department of Pathology Maastricht University Medical Center+ Maastricht The Netherlands

**Keywords:** basal cell carcinoma, imaging, optical coherence tomography

## Abstract

Optical coherence tomography (OCT), a non‐invasive diagnostic modality, may replace biopsy for diagnosing basal cell carcinoma (BCC) if a high‐confidence BCC diagnosis can be established. In other cases, biopsy remains necessary to establish a histopathological diagnosis and treatment regimen. It is, therefore, essential that OCT assessors have a high specificity for differentiating BCC from non‐BCC lesions. To establish high‐confidence BCC diagnoses, specific morphological BCC characteristics on OCT are used. This study aimed to review several cases of non‐BCC lesions that were misclassified as BCC by experienced OCT assessors, thereby providing insight into the causes of these misclassifications and how they may be prevented. The study population consisted of patients who had a histopathologically‐verified non‐BCC lesion. Patients from Maastricht University Medical Center+ from February 2021 to April 2021 were included in the study. Two independent OCT assessors assessed OCT scans. One OCT assessor recorded the presence or absence of validated morphological BCC characteristics. A false‐positive OCT test result was defined as certainty of BCC presence in a non‐BCC lesion. The frequency of misclassifications and the presence or absence of morphological BCC features are discussed. A total of 124 patients with non‐BCC lesions were included. Six patients were misclassified by both OCT assessors and are discussed in more detail. Histopathological diagnoses were squamous cell carcinoma (*n* = 2/21), actinic keratosis (*n* = 2/29), squamous cell carcinoma in situ/Bowen's disease (*n* = 1/16), or interphase dermatitis (*n* = 1/4). In all misclassified cases, multiple, apparent morphological BCC characteristics on OCT were present. Most non‐BCC lesions are recognized as such by OCT assessors. However, there remains a small risk that a high‐confidence BCC diagnosis is established in non‐BCC lesions wherein features mimicking validated BCC characteristics are present. Misclassification may be prevented by careful delineation of epidermal layers and good differentiation between dermal ovoid structures typical of BCC versus squamous cell carcinoma.

## 1 INTRODUCTION

In current clinical practice, the diagnosis of basal cell carcinoma (BCC) is regularly established using histopathological examination of biopsy specimens.^1^ However, non‐invasive diagnostic modalities, such as optical coherence tomography (OCT), are rapidly gaining popularity within the field of dermatology.^2^ It has been proposed that OCT may replace biopsy if OCT assessors are able to establish a BCC diagnosis with high confidence.^3^, ^4^ However, if non‐BCC lesions are incorrectly classified as BCC on an OCT scan, and directly treated without histopathological verification, patients may be at risk of receiving inadequate treatment. Therefore, the specificity of BCC diagnosis on OCT must be high.

Specific morphological BCC characteristics have been described and validated for diagnosing BCC on OCT.^5^, ^6^, ^7^ Adan et al.^7^ evaluated the diagnostic value of these morphological BCC characteristics on OCT and found that the characteristics with the highest diagnostic odds ratio (DOR) for discriminating BCC from non‐BCC lesions (DOR > 10) were atrophy of the epidermis, protrusions into the upper dermis with a dark rim, dermal dark rim, bright peritumoral stroma, and a shoal of fish/bunch of grapes appearance. The probability of a lesion with one or more discriminative characteristics being histopathologically verified BCC is high. In contrast, thickening of the epidermis appeared to be predictive of the absence of BCC.^7^ The DOR is a measure for the ability of a test to discriminate between subjects with and without a specific disorder.^8^ A DOR far above 1 indicates that the odds of having a BCC versus a non‐BCC is high, whereas a DOR far below 1 indicates that the odds of having a BCC versus a non‐BCC is low. The morphological BCC features on OCT with associated DORs are listed in Table 1.

**TABLE 1:** Morphological basal cell carcinoma features on optical coherence tomography and their respective diagnostic odds ratios as described in Adan et al.^7^

Feature	Diagnostic odds ratio	95% CI
Epidermis		
Protrusions into upper dermis with dark rim	15.18	6.66–34.59
Atrophy	12.11	4.27–34.32
Superficial scaling/crust/ulceration	0.92	0.54–1.56
Thickening	0.09	0.05–0.16
Dermal epidermal junction		
Dermal epidermal junction poorly defined/interrupted	9.17	4.98–16.88
Dermis		
Dark rim	64.11	27.02–152.11
Bright peritumoral stroma	49.38	20.09–121.40
Shoal of fish/bunch of grapes appearance	10.29	2.44–43.40
Hyporeflective ovoid structures	9.08	4.82–17.12
Black areas/cysts	9.00	2.73–29.64
Ovoid structures with bright center	5.61	2.16–14.53
Prominent vessels	1.06	0.63–1.80

Abbreviation: CI, confidence interval.

Previous studies have shown that experienced OCT assessors are well able to discriminate BCC from non‐BCC lesions. Most histopathological non‐BCC lesions are recognized as such with a specificity ranging from 93.9% to 96.0%.^3^, ^4^, ^9^ Nevertheless, there remains a small risk that a high‐confidence BCC diagnosis is made in non‐BCC lesions wherein specific BCC characteristics seem to be present.

The aim of this study was to review cases with non‐BCC lesions that were misclassified as BCC on OCT, and to provide more insight into the causes of such misclassifications and how they may be prevented in the future.

## 2 METHODS

The study population consisted of patients with a histopathological diagnosis of a non‐BCC lesion who participated in a recently published cohort study on the diagnostic accuracy of OCT assessment.^9^ Patients were enrolled from Maastricht University Medical Center+ from February 2021 to April 2021. Patients were ≥18 years old and had a lesion suspected to be non‐melanoma skin cancer or pre‐malignancy based on clinical and dermoscopic examination by their treating physician. Patient characteristics are described in Table 2. More details on the diagnostic cohort study are described elsewhere.^9^

**TABLE 2:** Patient and lesions characteristics of non‐basal cell carcinoma patients.

Characteristic	Total population (*N* = 124)
Age, mean SD, years	72 ± 11
Sex *n* (%)	
Men	68 (45)
Women	56 (55)
Localization *n* (%)	
Head or neck	67 (54)
Trunk	25 (20)
Extremities	32 (26)
Histopathological diagnosis *n* (%)	
Actinic keratosis	29 (23)
Bowen's disease	16 (13)
Squamous cell carcinoma	21 (17)
Interphase dermatitis	4 (3)
Other	54 (44)

Abbreviation: SD, standard deviation.

In brief, two OCT assessors (TW&FA) independently assessed OCT scans of whom one (TW) also recorded the presence or absence of specific morphological BCC characteristics as defined by Hussain et al.^5^ Both OCT assessors were blinded to the histopathological results and recorded their suspicion level for BCC presence. Certainty of BCC presence was considered a positive OCT test result, whereas uncertainty about BCC presence was considered a negative OCT test result. In the current study we describe the cases of non‐BCC lesions that were misclassified as BCC on OCT by both assessors. The specific OCT characteristics and histopathological findings of those misclassified lesions are discussed and the case with the highest quality OCT scan is described in more detail to illustrate the reasons for misclassification.

Optical coherence tomography scans were obtained using a Vivosight Multi‐beam Swept‐Source Frequency Domain OCT (Michelson Diagnostics; specifications: class 1 eye safe, resolution <7.5 μm lateral, <5 μm axial, depth of focus 1.0 mm, scan area 6 × 6 mm^2^). Histopathological results from a 3 mm punch biopsy of the scanned lesion were available for all patients and served as the reference standard for diagnosis. Histopathological examination of the biopsy specimens, embedded in paraffin and stained by hematoxylin‐eosin, was performed by experienced dermato‐pathologists, blinded to the OCT scans and assessments.

## 3 RESULTS

A total of 124 patients with non‐BCC lesions were included. Misclassifications by both OCT assessors occurred in six cases with either squamous cell carcinoma (SCC) (*n* = 2/21), actinic keratosis (AK) (*n* = 2/29), squamous cell carcinoma in situ/Bowen's disease (*n* = 1/16), or interphase dermatitis (*n* = 1/4). In all misclassified cases, multiple apparent morphological BCC characteristics on OCT were present.

### 3.1 Squamous cell carcinoma

Of the 21 histopathological SCCs included in the study, 19 (90.5%) were correctly classified as non‐BCC on OCT. A thickened epidermis was present in 18 of these 19 cases and is a strong negative predictor for BCC and a diagnostic feature of SCC.^7^, ^10^ In the one case without a thickened epidermis, no morphological BCC characteristics were present, therefore BCC could be ruled out. Morphological characteristics that are strong predictors for BCC presence were observed in only a few cases. Ovoid structures with bright centers or peritumoral hyperreflective stroma were detected only once (1/19) and dark cystic areas were detected three times (3/19).

Two of the 21 SCCs (9.5%) were misclassified as a BCC by both assessors. In both cases, hyporeflective ovoid nests, dermal dark rims, ovoid nests with bright centers, peritumoral hyperreflective stroma, and cystic areas were detected, which are strong positive predictors for BCC.^7^ In both misclassified cases, five or more morphological BCC characteristics were present, resulting in a high‐confidence BCC diagnosis on OCT.

#### 3.1.1 Case 1

**FIGURE 1:**
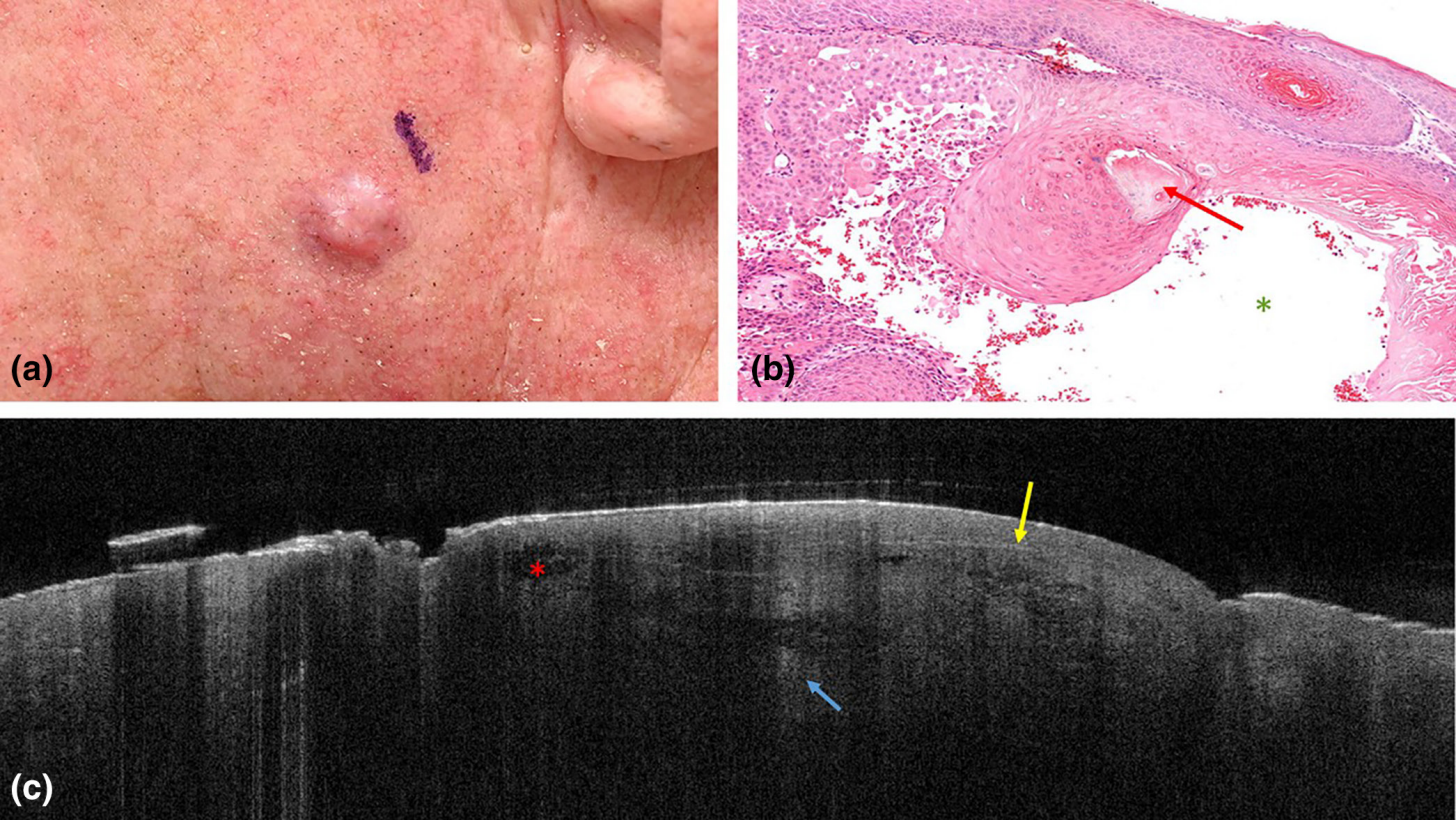
Clinical photograph of histopathology and optical coherence tomography scan of cutaneous squamous cell carcinoma on the left cheek of an 80‐year‐old man. (a) Shows the clinical presentation of the shiny plaque. (b) Shows a broadened epidermis and dermis which is fully infiltrated with squamous nests showing keratinization (red arrow) and cyst formation (green asterisk). Original magnification, ×130. (c) shows multiple tumor nests with varying reflectivity (yellow arrow). Areflective areas can be detected (red asterisk). Hyperreflective stroma surrounds the central tumor nests (blue arrow).

An 80‐year‐old man, with a medical history of multiple BCCs and one T1 SCC presented with a shiny plaque on his left cheek which had developed over the last 3 months (Figure 1a). The lesion grew, was sometimes painful, but did not itch or bleed. Dermoscopically, shiny white streaks were detected, and the treating physician was certain that the lesion was a nodular BCC.

Optical coherence tomography showed multiple large round hypo‐ and hyperreflective ovoid structures, which were thought to be ovoid BCC nests (Figure 1c). Some of these nests had areflective areas, which were classified as cystic ovoid nests. The structures were poorly demarcated, but the surrounding stroma was hyperreflective, which was thought to be peritumoral inflammation. Both OCT assessors classified the lesion as nodular BCC.

On histopathology, the epidermis was slightly thickened. In the dermis there were infiltrative nests of atypical keratinocytes. In multiple nests, keratinization was present. In addition, cyst formation was detected in some tumor nests (Figure 1b). The lesion was classified as a well‐differentiated, partially cystic, SCC.

### 3.2 Actinic keratosis

Of the 29 histopathological AK lesions that were included, 27 (93.1%) were correctly classified as non‐BCC on OCT. The most noticeable findings on OCT were confined to the epidermis. A thickened epidermis, which is a strong negative predictor for BCC and a diagnostic feature of AK, was present in 16 (59.3%) cases.^10^ On one scan (3.7%), protrusions with a dark rim were visible, which is a strong positive predictor for BCC, and more specifically for the superficial subtype.^7^ However, in this case misclassification did not occur, probably due to the presence of a thickened epidermis in other areas of the scan. Epidermal atrophy, which is a strong positive predictor for BCC, was absent in all 27 correctly identified AKs.

In the 2 out of 29 AKs (6.9%) misclassified as BCC by both assessors, a superficial subtype was suspected on OCT because protrusions with a dark rim were visible whereas thickening of the epidermis seemed absent. The suspicion for BCC was further reinforced by hyperreflective peritumoral stroma right under the protrusions with a dark rim artifact, which is a strong positive predictor for BCC.

#### 3.2.1 Case 2

**FIGURE 2:**
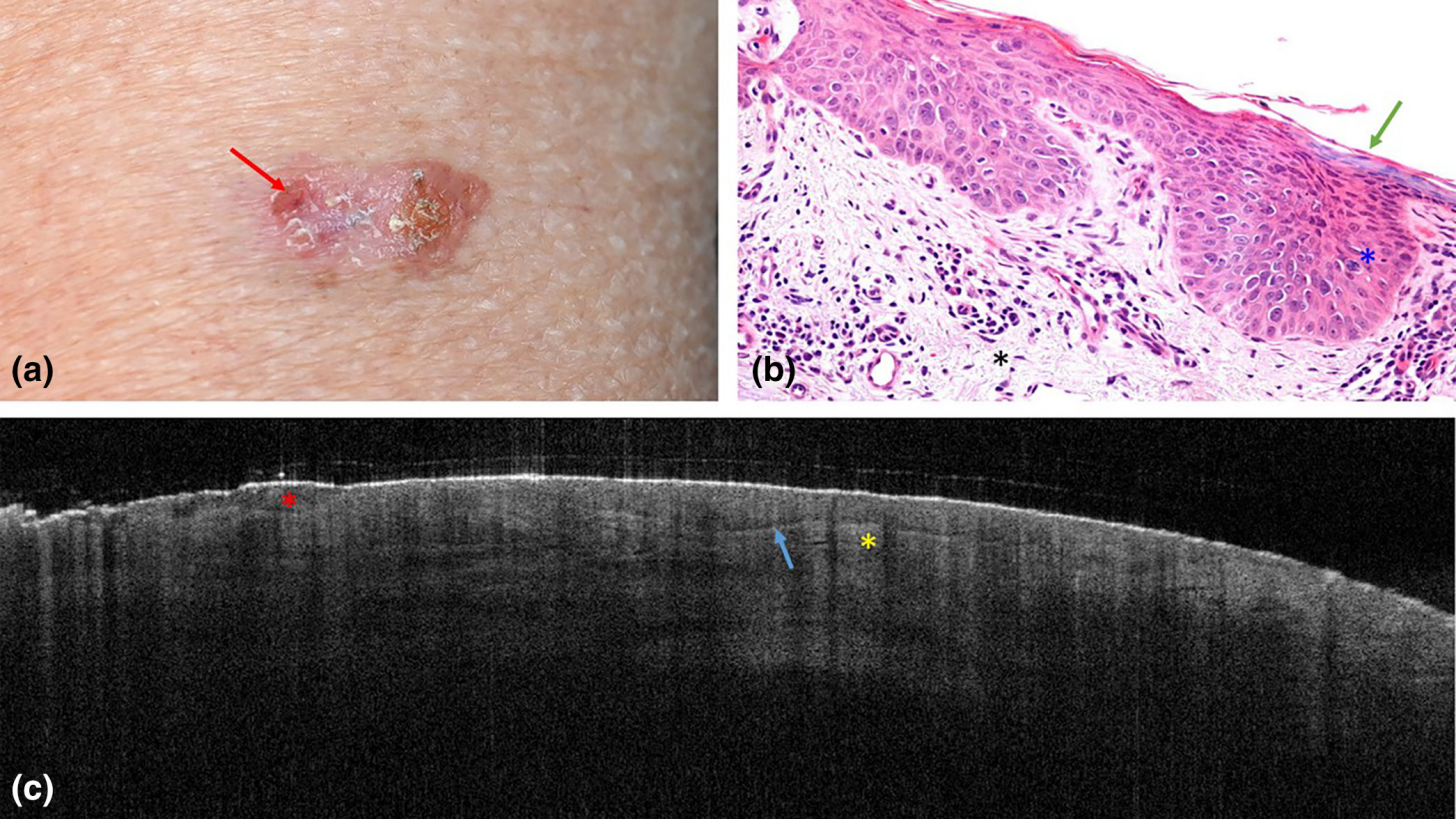
Clinical photograph histopathology and optical coherence tomography scan (of actinic keratosis on the left upper arm of an 81‐year‐old man. (a) Shows the clinical presentation of the erosive (red arrow) and erythematous mild keratotic plaque. (b) Shows atypical epidermal maturation with pleomorphic and hyperchromatic nuclei, and mitotic figures (blue asterisk) and parakeratosis (green arrow). No invasive growth was reported. The underlying dermis shows mild inflammation and solar elastosis (black asterisk). Original magnification, ×320. (c) Shows multifocal protrusions with a dark rim caused by the pleomorphic nuclei (blue arrow). Superficial erosions are detected (red asterisk). The dermal epidermal junction was focally poorly defined. The sub epidermal layer shows focal hyper reflectivity (yellow asterisk).

An 81‐year‐old man on systemic tacrolimus treatment after a kidney transplant 14 years ago, presented with a superficially erosive, erythematous, and mild keratotic plaque on the left upper arm (Figure 2a). The treating physician was certain of BCC presence but remained uncertain of the BCC subtype. A superficial subtype was considered most likely.

On OCT, epidermal protrusions with a dark rim were detected. Multiple superficial epidermal erosions correlated well with the clinical diagnosis superficial BCC. The dermal epidermal junction was poorly defined. In the papillary dermis, right under the protrusions with a dark rim, hyperreflective stroma was interpreted as peritumoral inflammation (Figure 2c). Both OCT assessors classified the lesion as superficial BCC.

On histopathology, there was epithelial dysmaturation with atypical keratinocytes that showed pleomorphic and hyperchromatic nuclei with multiple foci of mitotic cells. No cytonuclear atypia was found in the stratum granulosum. There was no invasive growth. In the underlying dermis, mild inflammation and solar elastosis were detected (Figure 2b). The lesion was classified as Bowenoid AK.

### 3.3 Squamous cell carcinoma in situ/Bowen's disease

Of the 16 histopathological cases of Bowen's Disease (BD) included in the study, 15 (93.8%) were correctly classified as non‐BCC on OCT. The most noticeable findings on OCT were confined to the epidermis. Epidermal thickening, which is a strong negative predictor for BCC and a diagnostic feature of BD, was present in 12 cases (80%).^10^ On one OCT scan (6.7%), protrusions with a dark rim were visible. However, misclassification did not occur, probably because of the unusual appearance of these protrusions, which seemed to merge into each other causing a slightly thickened epidermis. Epidermal atrophy was absent in all cases and no dermal morphological BCC characteristics were present besides prominent vessels in one case.

One out of 16 BDs (6.3%) cases was misclassified as a BCC by both assessors. What made this case particularly challenging was the presence of both epidermal and dermal morphological BCC characteristics. This was the only BD case in which protrusions with a dark rim, ovoid nests, and hyperreflective peritumoral stroma seemed present on OCT, which are all strong predictors for BCC.

#### 3.3.1 Case 3

**FIGURE 3:**
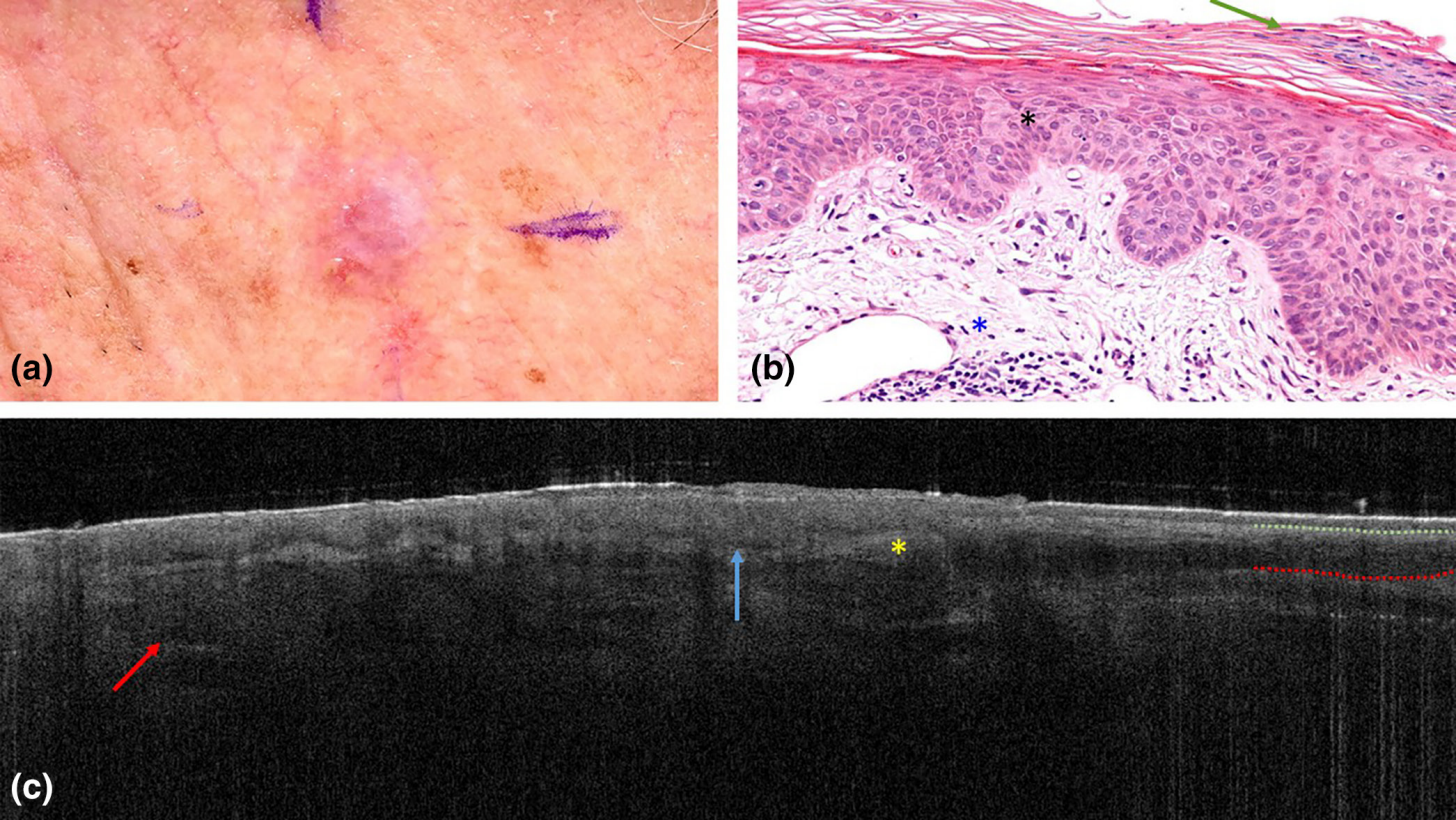
Clinical photograph of histopathology and optical coherence tomography scan of Bowen's disease (squamous cell carcinoma in situ) on the left cheek of a 79‐year‐old man. (a) Shows the clinical presentation of the erythematous shiny plaque. (b) Shows a slightly broadened epidermis with parakeratosis (green arrow). Pagetoid growth pattern (black asterisk) of keratinocytes with pleomorphic nuclei and abundant clear cytoplasm as well as mitotic figures can be seen. Inflammation and solar elastosis (blue asterisk) are detected in the dermis. Original magnification, ×320. (c) Shows apparent epidermal protrusions with a dark rim (blue arrow) and peritumoral hyperreflective stroma (yellow asterisk). The protrusions are located in the stratum corneum. The dermal epidermal junction is difficult to locate. The apparent dermal epidermal junction (green dotted line) demarcates the very hyporeflective stratum corneum and not an atrophic epidermis. The true dermal epidermal junction (red dotted line) resides deeper and demarcates a thickened epidermis. The dermis shows multiple well‐demarcated round hyporeflective structures mimicking ovoid nests (red arrow).

A 79‐year‐old man with a medical history of multiple BCCs and AK presented with an erythematous, slightly shiny plaque on his left cheek (Figure 3a). Dermoscopically, a homogenous white central area was detected with peripheral white streaks and some comma‐shaped vessels. Superficial BCC ranked high in the differential diagnosis, but AK or a benign verrucous lesion were also considered as possible diagnoses.

On OCT, the epidermis revealed multiple protrusions with a dark rim. The dermal epidermal junction seemed focally discontinued by the dark rims. Directly under the epidermis, hyperreflective stroma was detected, which was interpreted as peritumoral inflammation. In the dermis, multiple round hyporeflective ovoid structures were found with a dark peripheral border. The structures did not confluence with the epidermis and were classified as ovoid nests, even though they were not homogenous in terms of reflectivity (Figure 3c). Both OCT assessors classified the scan as BCC with a superficial and nodular subtype.

Histopathological examination of the lesion showed a slightly broadened epidermis with parakeratosis. Keratinocytes showed hyperchromatic and pleiomorphic nuclei with abundant clear cytoplasm and a pagetoid growth pattern as well as mitotic figures over the full epidermal width. The dermis showed solar elastosis and lymphocytic inflammation. Adnexal structures had a normal histomorphology (Figure 3b). The lesion was classified as BD.

### 3.4 Interphase dermatitis

Of the four cases with histopathological interphase dermatitis, three lesions (75%) were correctly identified as non‐BCC on OCT. None of these lesions had protrusions with a dark rim on OCT. Moreover, no dermal morphological BCC characteristics were detected besides prominent vessels, which seem to have a low value in discriminating BCC from non‐BCC.^7^

One of the four cases with interphase dermatitis (25%) was misclassified as a BCC by both assessors. In this case, protrusions with a dark rim were visible on OCT. The suspicion for a BCC was further reinforced by peritumoral hyperreflective stroma right under the epidermal protrusions with a dark rim. In addition, epidermal atrophy was present.^7^

#### 3.4.1 Case 4

**FIGURE 4:**
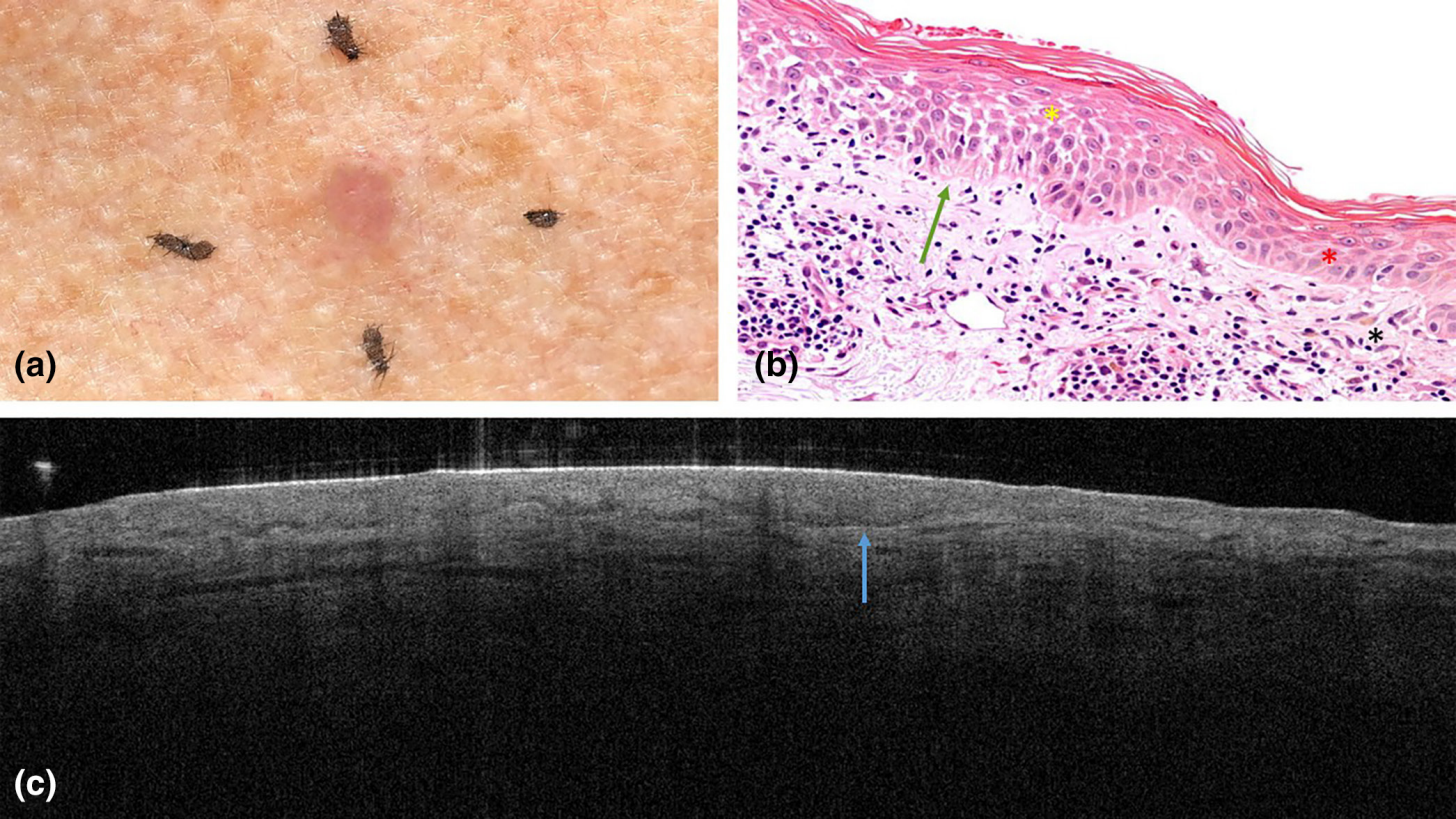
Clinical photograph, histopathology, and optical coherence tomography scan of interphase dermatitis (benign lichenoid keratosis) on the presternal area of a 57‐year‐old woman. (a) Shows the clinical presentation of the erythematous shiny papule. (b) Shows normal epidermis (red asterisk) transitioning to focal irregular broadening of the epidermis with spongiosis (yellow asterisk). The dermal epidermal junction shows vacuolization (green arrow). The dermis shows inflammation and mild pigment incontinence (black asterisk). Original magnification, ×420 (c) Shows multiple protrusions with a dark rim caused by vacuolization (blue arrow).

A 57‐year‐old woman presented with an erythematous shiny papule on the presternal area (Figure 4a). The recently detected lesion was slowly growing, pruritic, and did not bleed. Dermoscopically, there was no characteristic for BCC and hence BCC was low in the differential diagnosis. AK, benign lichenoid keratosis, and seborrheic keratosis were deemed likely diagnoses.

On OCT, multiple protrusions with a dark rim were detected. The dermal epidermal junction was poorly demarcated (Figure 4c). Both OCT assessors classified the lesion as superficial BCC.

Histopathological examination showed focal and irregular broadening of the epidermis with spongiosis in the basal layers. The dermal epidermal junction was focally poorly demarcated due to vacuolization. Within the dermis, perivascular and interstitial lymphocytic, and histiocytic infiltrations were detected as well as mild pigment incontinence (Figure 4b). The lesion was classified as interphase dermatitis, which clinically fits the diagnosis of benign lichenoid keratosis.

## 4 DISCUSSION

Based on the presence or absence of morphological BCC characteristics, most non‐BCC lesions can be well discriminated from BCC. However, in a few cases, morphological phenomena typical for other pathologies may mimic BCC characteristics on OCT, thereby contributing to a false‐positive BCC diagnosis on OCT. Therefore, continuous education on OCT assessment is essential and should not solely focus on the recognition of morphological BCC characteristics on OCT, but also on how these characteristics may be mimicked in non‐BCC lesions.

The level of reflectivity of OCT characteristics strongly depends on the absorption and scattering of the emitted light.^11^ Hence, morphological BCC characteristics may look slightly different in different lesions. Moreover, it is not only important to look at the presence of morphological BCC features, but also the context wherein they are present.

By differentiating apparent morphological BCC characteristics from true characteristics, misclassification of BCC may be prevented and high specificity can be preserved. For example, in all misclassified non‐BCC cases wherein a superficial BCC subtype was suspected on OCT (AKs, BD, and interphase dermatitis), apparent protrusions with a dark rim were visible on OCT. However, a focally thickened epidermis may easily be interpreted as a protrusion. Critical evaluation of which specific part of the epidermis is thickened aids in accurate differentiation between BCC and non‐BCC lesions. Thickening of the stratum corneum is uncommon in BCC, but may be observed in AK, BD, and SCC, and is usually induced by hyperkeratosis.^10^ Although stratum corneum thickening sometimes resembles a protrusion‐like pattern, it differs from protrusions that are indicative for BCC in that stratum corneum thickening is more hyporeflective and does not arise from the basal layer. Hence, hyporeflective protrusions arising from the stratum corneum are not indicative of BCC. By carefully differentiating the epidermal anatomical layers and locating the dermal epidermal junction this pitfall may be avoided. The lower boundary of the stratum corneum may mimic the dermal epidermal junction, leading to potential misinterpretation of the stratum corneum as an atrophic epidermis (Figure 3c). Therefore, it is essential to trace the apparent dermal epidermal junction until an undisputed epidermal layer is localized. If the apparent dermal epidermal junction aligns with the base of the hyperkeratosis, OCT assessors can determine that it is not the true anatomical dermal epidermal junction, because the latter must be located deeper. This reveals that the epidermis is not atrophic with protrusions but thickened with hyperkeratosis.

Some lesions show alternating epidermal thickening and atrophy which may be interpreted as protrusions on OCT. Possible causes are a pagetoid growth pattern, which may be detected in BD, or a spectrum of epidermal changes ranging from atrophic to hyperplastic in interphase dermatitis. It is, therefore, important to evaluate whether a protrusion has a dark rim. However, dysplastic cell content as found in BD, or vacuolization as found in interphase dermatitis may mimic dark rims. In such cases, a cellular resolution as provided by reflectance confocal microscopy^12^, ^13^ and line‐field confocal optical coherence tomography (LC‐OCT),^14^ may be more suitable to rule‐out BCC, based on their superior axial and lateral resolution.

Optical coherence tomography experts consider hyporeflective dermal ovoid structures a key feature for nodular BCC.^6^ However, ovoid nests may have a cystic phenotype causing areflective areas, or necrosis causing hyperreflective areas.^5^, ^10^, ^15^ Hence ovoid, nests may be variable in reflectivity. Ovoid dermal structures with varying reflectivity are also a criterion for diagnosing SCC on OCT.^16^ Differentiating tumor nests of SCC and BCC on conventional OCT, therefore, remains challenging.^16^ Various studies have proposed that dynamic OCT (D‐OCT), which visualizes vessel morphology based on speckle variance may be more suitable than conventional OCT for detecting SCC.^16^, ^17^ D‐OCT may visualize a diversified pattern of irregularly shaped and arranged vessels in SCC as opposed to centered and tumor island infiltrating vessels in BCC.^16^ Hence the addition of D‐OCT to conventional OCT may be beneficial for differentiating between BCC and SCC in cases wherein ovoid dermal structures with varying reflectivity are present.

Optical coherence tomography only provides information on the gross architecture of the skin. It is difficult to evaluate the contents of dermal ovoid structures, although this could be beneficial for differentiating BCC from non‐BCC. LC‐OCT and its superior resolution to OCT may provide more insight into the cellular components of dermal ovoid structures and various case reports have elucidated the potential of LC‐OCT assessment to differentiate dermal ovoid structures and rule‐out BCC.^18^, ^19^ However, the penetration depth of LC‐OCT is low (500 μm) and might be too limited to visualize tumor nests that grow deeper within the dermis.

This clinical series is limited by using only two experienced OCT assessors. Consequently, we chose to discuss false‐positive cases by both OCT assessors, yet other clinical imitators of BCC, such as sebaceous hyperplasia and dermal nevi, may be challenging to differentiate on OCT. Educational case reports and series may be beneficial to further improve the discriminative ability of OCT assessors.

Until now, training of OCT assessors has primarily focused on recognition of morphological BCC characteristics on OCT, with limited attention given to potential pitfalls when distinguishing BCC from non‐BCC lesions. Although OCT assessors correctly classify most non‐BCC lesions, there remains a small risk of misclassification, particularly when morphological BCC characteristics are observed on OCT scans of non‐BCC lesions. Misclassification may be prevented by careful delineation of epidermal layers and differentiation between dermal ovoid structures that are typical for BCC versus SCC. Differentiation of anatomical layers and ovoid structures may be enhanced by using D‐OCT or LC‐OCT.

## CONFLICT OF INTEREST STATEMENT

None declared.

## IRB APPROVAL STATUS

Reviewed and approved 16‐4‐197.1.

## PATIENT CONSENT

Patient consent forms are on file.

## Data Availability

Data will be made available on reasonable request.
